# Update: proposed reference sequences for subtypes of hepatitis E virus (species *Orthohepevirus A*)

**DOI:** 10.1099/jgv.0.001435

**Published:** 2020-05-29

**Authors:** Donald B. Smith, Jacques Izopet, Florence Nicot, Peter Simmonds, Shahid Jameel, Xiang-Jin Meng, Heléne Norder, Hiroaki Okamoto, Wim H.M. van der Poel, Gábor Reuter, Michael A. Purdy

**Affiliations:** ^1^​ Nuffield Department of Medicine, University of Oxford, Oxford, UK; ^2^​ INSERM, UMR1043, Toulouse F-31300, France; ^3^​ The Wellcome Trust/DBT India Alliance, Hyderabad, India; ^4^​ College of Veterinary Medicine, Virginia Polytechnic Institute and State University, Blacksburg, Virginia, USA; ^5^​ Department of Infectious Diseases, Institute of Biomedicine, Sahlgrenska Academy, University of Gothenburg, 41345 Gothenburg, Sweden; ^6^​ Division of Virology, Department of Infection and Immunity, Jichi Medical University School of Medicine, Tochigi-ken, Japan; ^7^​ Wageningen Bioveterinary Research, Wageningen University and Research, Lelystad, The Netherlands; ^8^​ Department of Medical Microbiology and Immunology, Medical School, University of Pécs, Pécs, Hungary; ^9^​ Centers for Disease Control and Prevention, National Center for HIV/Hepatitis/STD/TB Prevention, Division of Viral Hepatitis, Atlanta, Georgia, USA

**Keywords:** hepatitis E virus, *Hepeviridae*, *Orthohepevirus A*

## Abstract

In this recommendation, we update our 2016 table of reference sequences of subtypes of hepatitis E virus (HEV; species *Orthohepevirus A*, family *Hepeviridae*) for which complete genome sequences are available (Smith *et al*., 2016). This takes into account subsequent publications describing novel viruses and additional proposals for subtype names; there are now eight genotypes and 36 subtypes. Although it remains difficult to define strict criteria for distinguishing between virus subtypes, and is not within the remit of the International Committee on Taxonomy of Viruses (ICTV), the use of agreed reference sequences will bring clarity and stability to researchers, epidemiologists and clinicians working with HEV.

## Introduction

Hepatitis E virus (HEV) infects humans and a wide variety of other mammalian hosts. HEV is classified as a member of the species *Orthohepevirus A*, genus *Orthohepevirus* in the family *Hepeviridae* and comprises a number of genetically distinct genotypes, some of which can be divided further into subtypes. Previous analysis of genomic and subgenomic sequences has led to the recognition of four genotypes and 24 subtypes [1], which we recently updated to include seven genotypes and 31 subtypes, for which complete genome reference sequences were assigned for all but two subtypes [2]. We made a conservative decision to only recognize further subtypes when these were represented by complete coding region sequences from at least three epidemiologically unrelated virus sequences that were phylogenetically distinct from previous strains [2]. In addition, we were unable to provide consistent distance-based criteria for the assignment of complete genome sequences to subtypes. As a consequence of these two factors, 12 complete genome sequences were left unassigned to a subtype.

Genotypes and subtypes of HEV have been shown to be associated with host species [3] and geographical origin, while some studies, but not others, have found correlations with clinical outcome [4–6].

While the proposed genotype and subtype assignments have epidemiological and potential clinical value, it has remained problematic to produce consistent distance-based criteria for making these divisions, even when comparisons were limited to a particular genotype. We have therefore continued the practice of assigning sequences to subtypes according to their phylogenetic position. This paper updates this table and nomenclature by including proposed reference sequences for several additional subtypes.

## Methods

In total, 744 HEV sequences longer than 5000 nt using the search term {“hepatitis E virus” 5000 : 10000 [SLEN]} were downloaded from GenBank in February 2020 and manually aligned as amino acid sequences in SSEv1.4 [7]. After deleting non-virus sequences, sequences from viruses that were not members of the species *Orthohepevirus A*, sequences that did not include the complete coding region and the recombinant sequences D11092 [8], MG783571 [9], KJ013414, KJ013415 and KT633715 (J. Izopet and F. Nicot, unpublished results), and DQ450072 [8], 587 sequences remained (Fig. S1). From these were excised both the shared hypervariable region (amino acid positions 2119–2329 of ORF1 of M73218) and the subtype 3ra hypervariable regions (located in the subtype 3ra sequences at a position equivalent to ORF1 amino acids 2782–2783 of M73218). Nucleotide p-distances were calculated within SSE v1.4, and phylogenetic trees were produced based on maximum composite likelihood distances of complete coding sequences using mega7 [10]. Reference sequences for existing subtypes [2] were used to identify subtype clades. The alignment is available on the ICTV website https://www.ictv.global/ictv_wikis/hepeviridae/w/sg_hepe).

## Results

### Genotype 1

**Fig. 1.**
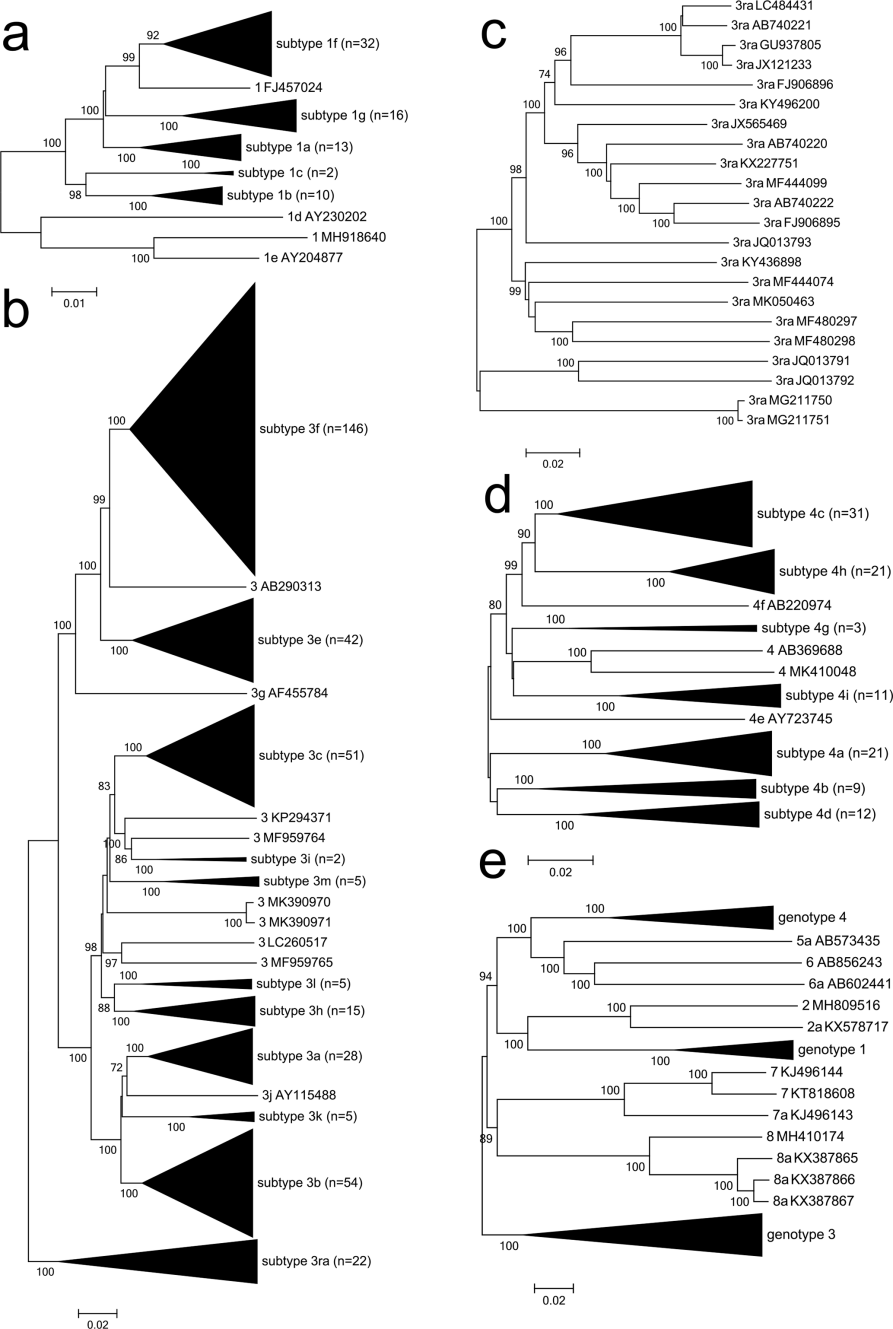
Phylogenetic analysis of HEV complete coding region sequences of (a) genotype 1 (*n*=77), (b) genotype 3 (*n*=384), (c) subtype 3ra (*n*=22), (d) genotype 4 (*n*=112) and (e) genotypes 5–8 and reference sequences of genotypes 1–4. Neighbour-joining trees of Jukes–Cantor distances was produced using mega7 [10]. Numbers indicate nodes where bootstrap support was >70 % out of 1000 replicates. Tips are collapsed for subtypes (panels a, b and d) or genotypes (panel e) for which multiple sequences are available. Bar: nucleotide sequence distances.

Nucleotide sequence p-distances among HEV genotype 1 complete genome sequences formed a continuous distribution up to 0.122. Distances within subtypes range up to 0.06, overlapping the range of inter-subtype distances (>0.037) including distances of 0.056–0.064 between subtypes 1b and 1c, and 0.044–0.082 between subtypes 1a and 1f. The overlap in ranges of inter- and intra-subtype comparisons was even more pronounced for amino acid distances. Hence, subtype assignments were made on the basis of their phylogenetic position (Fig. 1a) rather than p-distances.

A strain from Mongolia (MNE15-072, LC225387) is distinct from any of the genotype 1 reference subtype sequences, and has been proposed as representing a new subtype, 1g [11]. Several additional complete genome sequences closely related to this sequence are now available, from India (KY436505–7), Japan (LC314156, LC314158, and the more divergent sequences LC314155 and LC314157) [12], the UK (MH504155–6, MH504158–61, MH504163) and France (MN401238); together these sequences form subtype 1g with LC225387 as the reference sequence.

The Indian strain HEV-H (FJ457024) is most closely related to sequences of subtype 1f (p-distances of 0.037–0.058) and shares a branch with them on the phylogenetic tree, while NG/17-0503 from Nigeria (MH918640) is most closely related to subtype 1e (p-distance 0.0554). These two sequences are currently unassigned. All other strains fall into well-defined groups with previously described subtypes, although we note that several clades are present within subtypes 1a, 1f and 1g. The reference sequence for subtype 1b has been reassigned to L08816 because there is evidence that the original reference sequence D11092 is a recombinant [8].

### Genotype 2

Only three HEV genotype 2 complete sequences are available. M74506 and KX578717 both derive from the same stool sample from an outbreak in Mexico in 1986 and belong to subtype 2a [13, 14]. We have replaced M74506 with KX578717 as the genotype 2a reference sequence because, having being obtained more recently, this is likely to be the more accurate of the two, although the 5′- and 3′-untranslated regions are missing.

The sequence MH809516 was obtained from Nigeria in 2017 [15] and has a nucleotide p-distance from the 2a sequences of 0.156–0.158. This Nigerian sequence is most closely related to ORF2 sequences of Nigerian strains from a 1997 outbreak (AF172999–3001, AF173230–2) and individual strains from Chad (2004, AY903950) and Central Africa (2002, DQ151640), and more distantly related to strains from Namibia in 1995 (AY370686–9). Phylogenetic analysis of ORF2 sequences [15] reveals that MH809516 falls within a cluster of African strains previously assigned as subtype 2b [1]. MH809516 will become the reference sequence for subtype 2b once additional complete genome sequences become available.

### Genotype 3

Most HEV genotype 3 complete genome sequences fall into subtype groups as previously described, although there is considerable diversity within subtypes 3a, 3b, 3c, 3e, 3f and 3h. Nucleotide sequence distances within subtypes range up to 0.134 while distances lower than this are found for comparisons between subtypes 3a, 3b and 3k. Instead of imposing an arbitrary cut-off on a continuous distribution of sequence distances, we have grouped together sequences into one subtype where there are no gaps in the distribution of distances, and the group shares a common branch (Fig. 1b). We propose the following:

1. Sequences grouped into subtype 3f are extremely diverse and now include the previously unclassified sequences EU723513 and EU360977.
2. Sequences of the proposed subtype 3k (LC176492 and LC176493) [16] group with LC131066 and the previously unassigned sequences AB369689 and AB740232 and together become subtype 3k with AB369689 as the reference sequence.
3. Sequences of the proposed subtype 3l (MG674164 and KY766999) [17] group with JQ953664 and the sequences MF444121 and MF444131. All five are distinct from, but closest to, members of subtype 3h. Subtype 3l is added as a subtype with JQ953664 as the reference sequence.
4. Four sequences of the proposed subtype 3chi-new (MF444089, KU176130, KU513561 and MF444030) [18–20] fall into two groups, with the first three closely related and MF444030 more distant. We propose that this new subtype be called 3m, following previous naming conventions, with KU513561 as the reference sequence, being the first to be reported.
5. Four sequences from Switzerland (MF346772, MF346773, MG573193 and KY780957) proposed as subtype 3s [21, 22] share a common branch with eight closely related subtype 3h sequences and the more distantly related AB290312. Distances among all these sequences are all <0.125, within the range observed within subtype 3f (up to 0.132), and so these Swiss sequences are all considered as subtype 3h.
6. Sequences derived from rabbits (and closely related sequences from humans) form a phylogenetically distinct but diverse group (subtype 3ra, Fig. 1c). Depending on where a division is made, there would be from three to 14 subgroups. We have elected not to make an arbitrary division into additional subtypes, or sub-subtypes.
7. Several sequences (AB290313, MK390970, MK390971, LC260517, MF959765, MF959764 and KP294371) remain unclassified, being phylogenetically distinct from other subtypes but only represented currently by one or two sequences. It is striking that these unclassified genotype 3 sequences as well as the divergent 3h strains mentioned above (MG573193, MF346773 and AB290312) all derive from wild boar, pigs or pork products.

### Genotype 4

Nucleotide sequence p-distances among genotype 4 strains range up to 0.174 with no break in the distribution that could be used to consistently assign subtypes; a gap would exist from 0.117 to 0.121 were it not for comparisons between divergent subtype 4b sequences. The previously assigned subtypes 4a, 4d, 4g, 4h and 4i each comprise diverse sequences with a broad range of intra-subtype p-distances. Division into the existing subtypes has been preferred to the alternative in which each of these groupings would be split into multiple subtypes in an inconsistent way, because this would entail many of these additional subtypes sharing a long branch that was supported by bootstrap replication. A more ambiguous situation occurs for subtypes 4b and 4c where in each case a relatively short common branch leads to a radiation of three well-defined groups of sequences (represented by the subtype 4b sequences DQ279091, LC436450 and AB253420, and the subtype 4c sequences AB074915, AB161717 and AB220971). We have chosen to note this situation without splitting these subtypes up. The reference sequence for subtype 4i becomes AB369690 because there is evidence that the original reference sequence DQ450072 is a recombinant between subtype 3b, 4h and 4i sequences [8]. Sequences that lie outside these groups include 4e (AY723745), 4f (AB220974), AB369688 and MK410048 and derive from strains infecting both humans and pigs (Fig. 1d).

### Genotypes 5–8

In retrospect, HEV genotypes 5 and 6, with strains obtained from wild boar, might have been more consistently considered as subtypes of the same genotype, but there is little to be gained by changing the nomenclature now. No additional sequences are available for either genotype. Genotype 7 is represented by three sequences from dromedary camels that may represent two subtypes, but the second subtype has not been named as there are currently only two representatives (Fig. 1e). The sequences of HEV strains derived from Bactrian camels are phylogenetically distinct from all other genotypes [23, 24] and have been considered as genotype 8. Bootscan analysis suggests that genotype 8a is a recombinant between genotype 7a (ORF1) and genotype 1c (ORF2/3) sequences [25]. However, phylogenetic analysis of ORF2/3 of these sequences reveals a very deep branch between the genotype 8a and subtype 1c sequences, while scans of sequence distances between genotype 8a and other genotypes do not show evidence of recombination. We suggest that KX387865 should be considered as the reference sequence for genotype 8a, with MH410174 as an unclassified strain of genotype 8 (Fig. 1e). Similar relationships between genotypes 5–8 and genotypes 1–4 were observed when analysis was carried on individual ORFs as either nucleotide or amino acid sequences (Fig. S1).

## Discussion

We have updated the 2016 list of proposed reference sequences for subtypes of HEV (species *Orthohepevirus A*) by adding subtypes 1g, 3k, 3l, 3m and 8a (Table 1). Three major clades of genotype 3 subtypes are commonly referred to as genotype 3 group 1 (also known as Gt3-1, or HEV-3efg), genotype 3 group 2 (Gt3-2, or HEV-3abchij) and subtype 3ra. The additional subtypes 3k, 3l and 3m all belong to genotype 3 group 2. We have revised the reference sequences for subtypes 1b and 4i because there is evidence that the original reference sequences were recombinants.

**Table 1.** Reference complete genome sequences for HEV subtypes (bold – additions and alterations since 2016)

Subtype	Accession	Strain	Geographical origin*	Host*	Comments
1a	M73218	Burma	India, Pakistan, UK, Myanmar	Human	
**1b**	**L08816**	**Xinjiang**	China, Pakistan	Human	**Replaces D11092**
1c	X98292	I1	India	Human	
1d	AY230202	Morocco	Morocco	Human	
1e	AY204877	T3	Chad	Human	
1f	JF443721	IND-HEV- AVH5-2010	Bangladesh, India, UK	Human	
**1g**	**LC225387**	**MNE15-072**	India, Mongolia, Pakistan Japan, UK, France	Human	
1	**FJ457024**	**HEV-H**	India	Human	
1	**MH918640**	**NG/17–0503**	Nigeria	Human	
2a	**KX578717**	**Mex-14**	Mexico	Human	**Replaces M74506**
**2b**	(**MH809516**)	(**NG/17–0500**)	Nigeria	Human	(**Provisional**)
3a	AF082843	Meng	Japan, USA, South Korea, UK, Germany, Canada, Singapore, China, Mexico, Thailand	Human, pig, mongoose	
3b	AP003430	JRA1	Japan, China, Canada	Human, pig, wild boar, deer, mongoose	
3c	FJ705359	wbGER27	France, Germany, Netherlands, Sweden, UK, Thailand, Canada	Human, wild boar	
3d					AF296165-7 (ORF2)
3e	AB248521	swJ8-5	France, Germany, Hungary, Italy, UK, Japan	Human, pig, wild boar, *Maccaca* sp. monkey†	
3f	AB369687	E116-YKH98C	France, Germany, UK, Sweden, Denmark, Spain, Thailand, Japan, Singapore	Human, pig, wild boar	**Includes EU723513, EU360977 and KJ873911**
3g	AF455784	Osh 205	Kyrgyzstan	Pig†	
3h	JQ013794	TR19	France, Switzerland, Mongolia	Human, pig	
3i	FJ998008	BB02	Sweden, Germany	Human, wild boar	
3j	AY115488	Arkell	Canada	Pig	From pooled material
**3k**	**AB369689**	**E088-STM04C**	Japan	Human, pig	
**3l**	**JQ953664**	**FR-SHEV3c-like**	Italy, France	Pig, human	
**3m**	**KU513561**	**IC2011**	France, Spain	Human	
3	AB290313	swMN06-C1056	Mongolia	Pig	
**3**	**MF959765**	**WB/HEV/** **NA21ITA15**	Italy	Wild boar	
**3**	**LC260517**	**swHE1606845**	Japan	Pig	
**3**	**MK390971**	**17RS1920**	Italy	Wild boar	
**3**	**MF959764**	**WB/HEV/** **NA17ITA15**	Italy	Wild boar	
**3**	**KP294371**	**MWP_2010**	Germany	Wild boar	
**3ra**	**FJ906895**	**GDC9**	France, Germany, China, South Korea, USA	Rabbit, hare, human	Diverse clade
4a	AB197673	JKO-ChiSai98C	China, Mongolia, Taiwan, South Korea	Pig, human	
4b	DQ279091	swDQ	Taiwan, China, Cambodia, Japan	Pig, human, Rhesus monkey	Three subclades
4c	AB074915	JAK-Sai	Japan	Human, pig	Three subclades
4d	AJ272108	T1	China	Pig, human	
4e	AY723745	IND-SW-00–01	India	Pig	
4f	AB220974	HE-JA2	Japan	Human	
4g	AB108537	CCC220	Japan, China	Human	
4h	GU119961	CHN-XJ-SW13	China	Human, pig, cow, goat, yak	
**4i**	**AB369690**	**E067-SIJ05C**	China, Japan	Pig, wild boar human	**Replaces DQ450072**
4	AB369688	E087-SAP04C	Japan†	Human	
**4**	**MK410048**	**SWU/42-5/2018**	China	Pig	
5a	AB573435	JBOAR135-Shiz09	Japan	Wild boar	
6a	AB602441	wbJOY_06	Japan	Wild boar	
6	AB856243	wbJNN_13	Japan	Wild boar	
7a	KJ496143	178C	UAE	Camel	Dromedary camel
7	KJ496144	180C	UAE	Camel, human	Dromedary camel
**8a**	**KX387865**	**12XJ**	China	Camel	**Bactrian camel**
**8**	**MH410174**	**BcHEV-GP**	China	Camel	**Bactrian camel**

*Information for strains for which a complete coding region sequence is available.

†Geographical origin or host uncertain.

While HEV genotypes are phylogenetically distinct, accurate assignment of strains to particular subtypes continues to be problematic because of the overlapping distributions of pairwise p-distances observed for inter- and intra-subtype comparisons. One way of addressing this problem is to automate the assignment process by partitioning phylogenetic trees based upon the observed distribution of distances among known members of a subtype [20]. However, not all sequences can be assigned by this method, and some assignments differ from the phylogeny-based ones given here.

An accessible and standard methodology for subtype allocation is important so that researchers can assign strains to particular subtypes. Online tools that have recently become available for the genotyping and subtyping of HEV sequences include HEV-GLUE (http://hev.glue.cvr.ac.uk) [26] and HEVnet (https://www.rivm.nl/mpf/typingtool/hev/) [27]. These tools can be used to make subtype assignments based on the analysis of HEV subgenomic sequences. However, it is important to note that these methods rely upon prior decisions having been made about the boundaries of subtype clades.

We have chosen to use a simple method of sequence analysis (phylogenetic analysis of nucleotide distances of complete genome sequences) using freely available software (mega7) [10] so that the analyses our decisions are based on will be reproducible and extendable by others in the field. We have also adopted a conservative approach to subtype definitions in order to avoid disruption to existing categories. We have also chosen to adhere to a sequential alphabetical schema for subtype naming, with the exception of the unusually divergent, rabbit-associated, subtype 3ra. These recommendations will be updated in 4 years’ time, or sooner if necessitated by new information.

Given the difficulty of defining consistent criteria for distinguishing subtypes of HEV, it is important that these subtypes are not simplistically treated as discrete categories. Strains within a subtype may be as divergent from each other as they are from strains in other subtypes. In addition, within a subtype there may be clades of closely related sequences, as well as more distantly related sequences belonging to the same subtype. When virus variation is being related to epidemiological, biological or clinical features of infection, it will be important to study the actual phylogenetic relationships that exist between sequences rather than treating subtype assignments as a defining characteristic.

## Supplementary Data

Supplementary material 1
