# Supplementary material for: Update: proposed reference sequences for subtypes of hepatitis E virus (species Orthohepevirus A)
Source: J Gen Virol. 2020 May 29;101(7):692–8. doi: 10.1099/jgv.0.001435 (PMC7660235; doi:10.1099/jgv.0.001435)
Supplement: Supplementary material 1 [file jgv-101-692-s001.pdf]

Supplementary file 1

List of hepatitis E virus sequences analysed with genotype and subtype designation.

1_FJ457024, 1_MH918640, 1a_AF051830, 1a_AF076239, 1a_AF185822, 1a_AF459438, 1a_D10330, 1a_DQ459342, 1a_JF443718, 1a_JF443719, 1a_JF443720, 1a_LC061267, 1a_M73218, 1a_MH504157, 1a_X99441, 1b_AF444002, 1b_AF444003, 1b_D11093, 1b_JQ655734, 1b_L08816, 1b_L25547, 1b_L25595, 1b_M80581, 1b_M94177, 1c_JF443717, 1c_X98292, 1d_AY230202, 1e_AY204877, 1f_AB720034, 1f_AB720035, 1f_JF443721, 1f_JF443722, 1f_JF443723, 1f_JF443724, 1f_JF443725, 1f_JF443726, 1f_MH074880, 1f_MH504154, 1f_MH504162, 1f_MH991993, 1f_MH991994, 1f_MH991995, 1f_MH991996, 1f_MH991997, 1f_MH991998, 1f_MH991999, 1f_MH992000, 1f_MH992001, 1f_MH992002, 1f_MH992003, 1f_MH992004, 1f_MH992005, 1f_MH992006, 1f_MH992007, 1f_MH992008, 1f_MH992009, 1f_MH992010, 1f_MH992011, 1f_MH992012, 1f_MH992013, 1g_KY436505, 1g_KY436506, 1g_KY436507, 1g_LC225387, 1g_LC314156, 1g_LC314158, 1g_MH504155, 1g_MH504156, 1g_MH504158, 1g_MH504159, 1g_MH504160, 1g_MH504161, 1g_MH504163, 1g_MN401238, 1g_LC314155, 1g_LC314157, 2_MH809516, 2a_KX578717, 2a_M74506, 3_AB290313, 3_KP294371, 3_LC260517, 3_MF959764, 3_MF959765, 3_MK390970, 3_MK390971, 3a_AB074918, 3a_AB074920, 3a_AB089824, 3a_AB481228, 3a_AB591734, 3a_AB630970, 3a_AF060668, 3a_AF060669, 3a_AF082843, 3a_AY575859, 3a_FJ426403, 3a_FJ426404, 3a_HW532736, 3a_HW532737, 3a_JN564006, 3a_JN837481, 3a_JQ679013, 3a_JQ679014, 3a_KF303502, 3a_KJ507955, 3a_KT447526, 3a_KT447528, 3a_KT727028, 3a_KX574712, 3a_KX981911, 3a_MG833836, 3a_MH450022, 3a_MH504130, 3b_AB073912, 3b_AB091394, 3b_AB189070, 3b_AB189071, 3b_AB189072, 3b_AB189073, 3b_AB189074, 3b_AB189075, 3b_AB222182, 3b_AB222183, 3b_AB222184, 3b_AB236320, 3b_AB246676, 3b_AB291951, 3b_AB291952, 3b_AB291953, 3b_AB291954, 3b_AB291955, 3b_AB291956, 3b_AB291957, 3b_AB291960, 3b_AB291962, 3b_AB291963, 3b_AB301710, 3b_AB362839, 3b_AB362840, 3b_AB362841, 3b_AB362842, 3b_AB362843, 3b_AB369691, 3b_AB425830, 3b_AB425831, 3b_AB437316, 3b_AB437317, 3b_AB437318, 3b_AB437319, 3b_AB443623, 3b_AB443624, 3b_AB443625, 3b_AB443626, 3b_AB443627, 3b_AB481229, 3b_AB591733, 3b_AB593690, 3b_AB630971, 3b_AB698071, 3b_AP003430, 3b_FJ527832, 3b_KJ507956, 3b_LC126331, 3b_LC126332, 3b_LC386855, 3b_LC439299, 3b_LC490579, 3c_FJ705359, 3c_KC618402, 3c_KC618403, 3c_KJ701409, 3c_KT159771, 3c_KU176129, 3c_KX172133, 3c_KX462160, 3c_MF444031, 3c_MF444042, 3c_MF444043, 3c_MF444044, 3c_MF444049, 3c_MF444063, 3c_MF444064, 3c_MF444065, 3c_MF444071, 3c_MF444072, 3c_MF444085, 3c_MF444106, 3c_MF444111, 3c_MF444114, 3c_MF444115, 3c_MF444122, 3c_MF444128, 3c_MF444143, 3c_MG783569, 3c_MG783570, 3c_MH377722, 3c_MH377723, 3c_MH377727, 3c_MH450021, 3c_MH504124, 3c_MH504125, 3c_MH504126, 3c_MH504127, 3c_MH504128, 3c_MH504129, 3c_MH504131, 3c_MH504132, 3c_MH504133, 3c_MH504134, 3c_MH504135, 3c_MH504136, 3c_MH504137, 3c_MH504138, 3c_MK089849, 3c_MN614139, 3c_MN614140, 3c_MN614141, 3c_MN629976, 3e_AB248520, 3e_AB248521, 3e_AB248522, 3e_AB291958, 3e_AB481226, 3e_AB780450, 3e_AB780451, 3e_AB780452, 3e_AB780453, 3e_FJ998015, 3e_HM055578, 3e_JQ013795, 3e_JQ026407, 3e_JQ953665, 3e_KF922359, 3e_KP698919, 3e_MF444086, 3e_MF444109, 3e_MF444141, 3e_MH184579, 3e_MH184580, 3e_MH184581, 3e_MH184582, 3e_MH184583, 3e_MH184584, 3e_MH377724, 3e_MH504139, 3e_MH504140, 3e_MH504141, 3e_MH504142, 3e_MH504143, 3e_MH504144, 3e_MH504145, 3e_MH504146, 3e_MH504148, 3e_MH504149, 3e_MH504150, 3e_MH504152, 3e_MH504153, 3e_MK089848, 3e_MN646690, 3e_MN646691, 3f_AB291961, 3f_AB369687, 3f_AB850879, 3f_EU360977, 3f_EU375463, 3f_EU495148, 3f_EU723512, 3f_EU723513, 3f_EU723514, 3f_EU723515, 3f_EU723516, 3f_FJ653660, 3f_FJ956757, 3f_JN906974, 3f_JN906975,

3f_JN906976, 3f_JQ953666, 3f_KC166967, 3f_KC166968, 3f_KC166969, 3f_KC166970, 3f_KC166971, 3f_KT447527, 3f_KT581443, 3f_KT581444, 3f_KT581445, 3f_KT581446, 3f_KT581447, 3f_KT581448, 3f_KT591532, 3f_KT591533, 3f_KT591534, 3f_KU747141, 3f_KU747142, 3f_KU980235, 3f_KY232312, 3f_KY232313, 3f_LC055972, 3f_LC055973, 3f_LC164712, 3f_MF444027, 3f_MF444028, 3f_MF444029, 3f_MF444032, 3f_MF444033, 3f_MF444034, 3f_MF444035, 3f_MF444036, 3f_MF444038, 3f_MF444039, 3f_MF444040, 3f_MF444041, 3f_MF444045, 3f_MF444046, 3f_MF444047, 3f_MF444048, 3f_MF444050, 3f_MF444051, 3f_MF444052, 3f_MF444053, 3f_MF444054, 3f_MF444055, 3f_MF444057, 3f_MF444058, 3f_MF444059, 3f_MF444060, 3f_MF444061, 3f_MF444062, 3f_MF444066, 3f_MF444067, 3f_MF444068, 3f_MF444069, 3f_MF444070, 3f_MF444073, 3f_MF444075, 3f_MF444076, 3f_MF444078, 3f_MF444079, 3f_MF444080, 3f_MF444081, 3f_MF444082, 3f_MF444083, 3f_MF444084, 3f_MF444087, 3f_MF444088, 3f_MF444090, 3f_MF444091, 3f_MF444092, 3f_MF444093, 3f_MF444094, 3f_MF444095, 3f_MF444096, 3f_MF444097, 3f_MF444098, 3f_MF444100, 3f_MF444101, 3f_MF444102, 3f_MF444103, 3f_MF444104, 3f_MF444105, 3f_MF444107, 3f_MF444108, 3f_MF444112, 3f_MF444113, 3f_MF444116, 3f_MF444117, 3f_MF444118, 3f_MF444119, 3f_MF444123, 3f_MF444124, 3f_MF444125, 3f_MF444126, 3f_MF444127, 3f_MF444129, 3f_MF444130, 3f_MF444132, 3f_MF444133, 3f_MF444134, 3f_MF444135, 3f_MF444137, 3f_MF444138, 3f_MF444139, 3f_MF444140, 3f_MF444142, 3f_MF444144, 3f_MH377725, 3f_MH377726, 3f_MH450020, 3f_MH450023, 3f_MH450024, 3f_MH450025, 3f_MH450026, 3f_MH450027, 3f_MH450028, 3f_MH450029, 3f_MH450030, 3f_MH450031, 3f_MH504147, 3f_MH504151, 3f_MK089847, 3f_MN401237, 3f_MN646689, 3f_MN646692, 3f_MN646693, 3f_MN646695, 3f_MN646696, 3g_AF455784, 3h_AB290312, 3h_JQ013794, 3h_KU176131, 3h_KU176132, 3h_KY780957, 3h_MF346772, 3h_MF346773, 3h_MF444037, 3h_MF444056, 3h_MF444077, 3h_MF444110, 3h_MF444120, 3h_MF444136, 3h_MF444145, 3h_MG573193, 3i_FJ998008, 3i_MH377721, 3j_AY115488, 3k_AB369689, 3k_AB740232, 3k_LC131066, 3k_LC176492, 3k_LC176493, 3l_JQ953664, 3l_KY766999, 3l_MF444121, 3l_MF444131, 3l_MG674164, 3m_KU176130, 3m_KU513561, 3m_MF444030, 3m_MF444089, 3m_MN646694, 3ra_LC484431, 3ra_AB740220, 3ra_AB740221, 3ra_AB740222, 3ra_FJ906895, 3ra_FJ906896, 3ra_GU937805, 3ra_JQ013791, 3ra_JQ013792, 3ra_JQ013793, 3ra_JX121233, 3ra_JX565469, 3ra_KX227751, 3ra_KY436898, 3ra_KY496200, 3ra_MF444074, 3ra_MF444099, 3ra_MF480297, 3ra_MF480298, 3ra_MG211750, 3ra_MG211751, 3ra_MK050463, 4_AB369688, 4_MK410048, 4a_AB197673, 4a_AB197674, 4a_EF077630, 4a_EU366959, 4a_FJ763142, 4a_GU119960, 4a_HQ634346, 4a_JQ655733, 4a_KC492825, 4a_KC692453, 4a_LC037955, 4a_MF567575, 4a_MK330953, 4a_MK410045, 4a_MK410046, 4a_MK410047, 4a_MK410049, 4a_MK410050, 4a_MK410051, 4a_MK410052, 4a_MK410053, 4b_AB253420, 4b_AB291964, 4b_DQ279091, 4b_EU676172, 4b_JX855794, 4b_LC042232, 4b_LC428039, 4b_LC436449, 4b_LC436450, 4c_AB074915, 4c_AB074917, 4c_AB080575, 4c_AB091395, 4c_AB097811, 4c_AB097812, 4c_AB099347, 4c_AB161717, 4c_AB161718, 4c_AB161719, 4c_AB193176, 4c_AB193177, 4c_AB193178, 4c_AB200239, 4c_AB220971, 4c_AB220972, 4c_AB220973, 4c_AB220975, 4c_AB220976, 4c_AB220977, 4c_AB220978, 4c_AB220979, 4c_AB291959, 4c_AB291965, 4c_AB291966, 4c_AB291967, 4c_AB291968, 4c_AB480825, 4c_AB481227, 4c_LC022745, 4c_LC387631, 4d_AJ272108, 4d_AY594199, 4d_FJ610232, 4d_GU206559, 4d_GU361892, 4d_HM152568, 4d_JQ655736, 4d_KC163335, 4d_KF176351, 4d_KX531115, 4d_KX827238, 4d_MK410044, 4e_AY723745, 4f_AB220974, 4g_AB108537, 4g_AB698654, 4g_LC387632, 4h_GU119961, 4h_GU188851, 4h_JQ655735, 4h_JQ740781, 4h_KF736234, 4h_KJ155502, 4h_KM253769, 4h_KR872414, 4h_KR872415, 4h_KR872416, 4h_KR872417, 4h_KU356182, 4h_KU356183, 4h_KU356184, 4h_KU356185, 4h_KU356186, 4h_KU356187, 4h_KU356188, 4h_KU356189, 4h_KY929403, 4h_KY929404, 4i_AB369690, 4i_AB521805,

4i_AB521806, 4i_AB602439, 4i_AB602440, 4i_AB909124, 4i_AB909125, 4i_EF570133, 4i_HM439284, 4i_JF915746, 4i_JQ993308, 5a_AB573435, 6_AB856243, 6a_AB602441, 7_KJ496144, 7_KT818608, 7a_KJ496143, 8a_KX387865, 8a_KX387866, 8a_KX387867, 8_MH410175, 8_MH410176, 8_MH410174,

Supplementary Figure 1

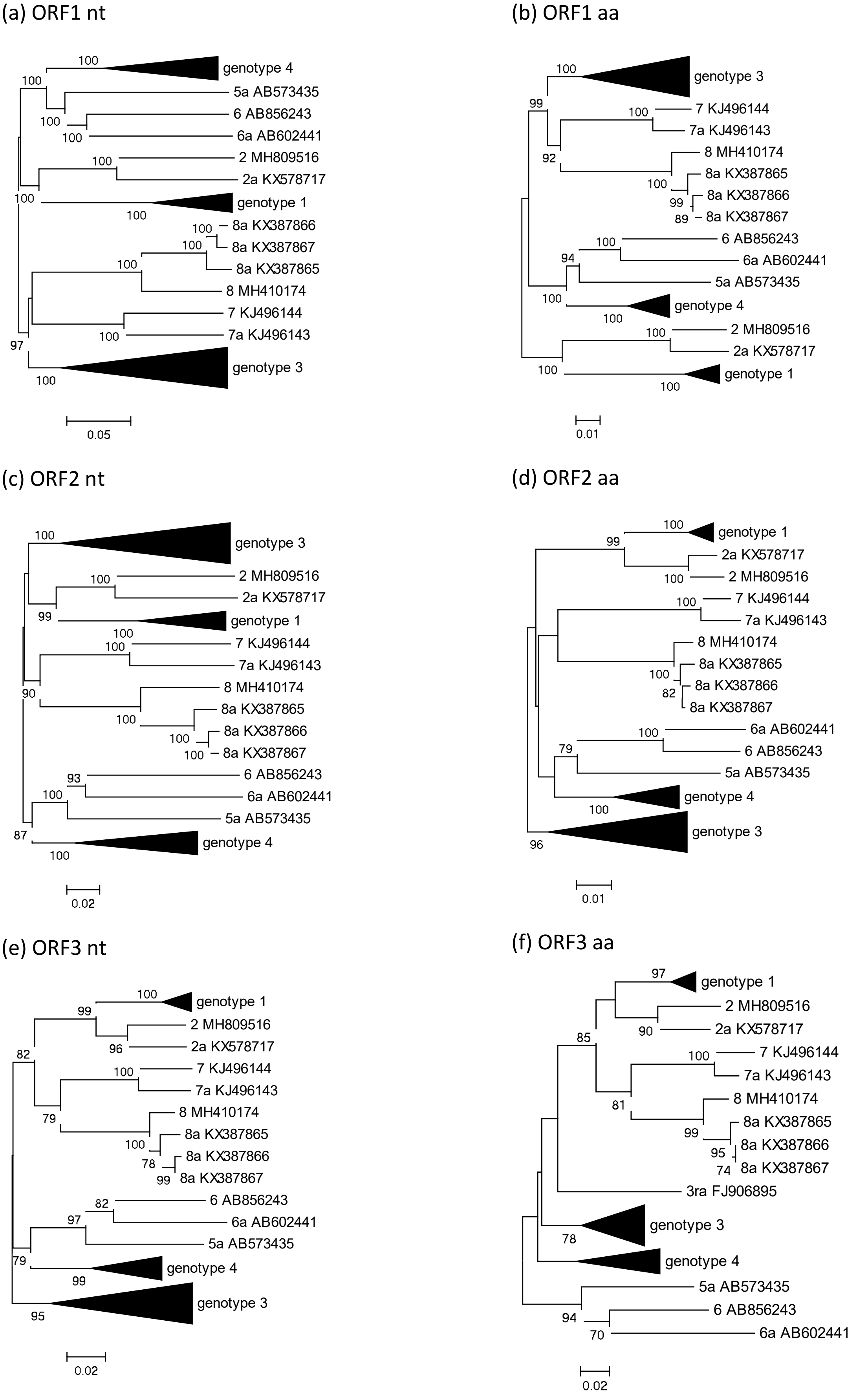

Figure legend Supplementary Figure 1

Phylogenetic analysis of hepatitis E virus ORFs 1–3. Aligned nucleotide (a, c, e) or amino acid (b, d, f) sequences of ORF1 (a, b), ORF2 (c, d) or ORF3 (e, f) of HEV reference strains as detailed in Table 1 were used to produced neighbour-joining phylogenetic trees using MEGA7 (Kumar *et al*., 2016). Trees were based on Jukes-Cantor distances (nucleotide sequences) or p-distances (amino acid sequences). The branches for genotypes 1, 3 and 4 have been collapsed. Numbers indicate bootstrap support where this was >70% (1000 replicates).
